# Detection and genotypes of piroplasms affecting ruminants in the New Valley Governorate, Egypt

**DOI:** 10.1186/s12917-025-05101-3

**Published:** 2025-11-15

**Authors:** Safaa Mohamed Barghash, Tarek Ramadan Abou Elnaga, Wafaa Abd-El Latif Osman, Mohamed Said Farrag, Sara Samy AlAsrag, Eman Abd El Tawab Noaman, Samah ElSayed Yassin

**Affiliations:** 1 Parasitology Unit, Animal and Poultry Health Department, Desert Research Center, Cairo, Egypt; 2 Diseases and Infectious Diseases Unit, Animal and Poultry Health Department, Desert Research Center, Cairo, Egypt; 3 Toxicology and Environmental Pollution Diseases Unit, Animal and Poultry Health Department, Desert Research Center, Cairo, Egypt

**Keywords:** Babesia, Theileria annulata, Ruminants, PCR, Gene sequencing, Egypt

## Abstract

**Background:**

Piroplasms, which include the two genera *Babesia* and *Theileria*, are protozoan parasites transmitted by Ixodid ticks that infect the erythrocytes of vertebrate hosts, including humans, domestic animals, and wild animals. The present study examined how common and distinct genotypes of the above tick-borne parasites are in the New Valley Governorate in Egypt, which covers 440,098 km².

**Materials and methods:**

It was conducted on 321 randomly selected live animals (89 cattle, 55 goats, and 177 sheep), regardless of sex and age. Of these, 203 were found to be infested with ticks, and 269 ticks were collected to determine the prevalent tick species microscopically. Giemsa-stained blood films and conventional polymerase chain reaction (cPCR) assays targeted the *Babesia 18 S rRNA gene* for Babesia species and the *T. annulata tams1 gene* for *T. annulata*, used for the detection of piroplasms. Then, we sequenced the eleven highest positivity-generated bands (6 for Babesia and 5 for *T. annulata*), performed a phylogenetic analysis on them, and submitted their data to the GenBank database.

**Results:**

The infestation rate was 63.2%, and three tick species were identified. *Rhipicephalus annulatus* was the most common tick species on cattle (67.7%), followed by *Hyalomma excavatum* (17.8%) and *Rhipicephalus sanguineus* (14.5%), which preferred sheep and goats. Babesia has been detected in 22.12%, *T. annulata* in 16.5%, and mixed infections in 7.79% of samples under a microscope. The percentages increased by PCR to 32.7% for Babesia and 22.1% for *T. annulata*, with mixed infections in 13.4%. Molecular analyses confirmed four Babesia subspecies introduced in the GenBank database under accession numbers PP892244, PP892245 (as *B. bigemina*), PP892249 (as *B. motasi*), PP892246 (as *B. bovis*), and PP892247, PP892248 (as *B. ovis*). Their identities to GenBank references range from 71.3% to 100% with divergence from 0.0 to 24. Whereas the five submitted isolates of *T. annulata* were distributed into two clades within a cluster (one contained PP894805, PP894806, and PP894807), and the other contained PP894808 and PP894809. Their identities range from 98.4 to 100%, with a divergence of 0.0 to 1.6 between each other and from 0.0 to 8.0 from others in GenBank.

**Conclusions:**

This study reveals that *T. annulata* were genetically identical to the other isolates from other continents with no significant genotypic differences between them, contrary to the *Babesia* spp., which were found to belong to four different subspecies and scattered across different clades.

**Supplementary Information:**

The online version contains supplementary material available at 10.1186/s12917-025-05101-3.

## Introduction

Hard ticks are blood-feeding ectoparasites that infest humans and animals and are vectors of pathogenic microorganisms that cause severe infectious diseases, including the piroplasm protozoa *Babesia* and *Theileria*, which are the focus of this study [1]. The family Ixodidae (hard ticks) is a large family with a worldwide distribution, of which 44 species have been identified. It taxonomically includes four genera: *Amblyomma*, *Hyalomma*, *Haemaphysalis*, and *Rhipicephalus* [2]. Accurate taxonomy of tick species is crucial for controlling tick-borne diseases and developing ecological and public health measures to manage health threats in the world [1]. Tick infestations in ruminants’ flocks have been surveyed in Egyptian deserts, including the Northwestern coastal region; Sinai Peninsula; the Southeastern desert; the border governorates; and the Swiss Canal governorates [1, 3–11]. Hyalomma spp. are the most successful desert-adapted tick species in Egypt. *H. dromedarii*, *H. impletatum*, *H. anatolicum excavatum*, and *H. marginatum marginatum* prefer camels but also parasitize cattle, sheep, goats, and horses [1]. *Boophilus annulatus* typically feeds on cattle [1, 12]. *Rhipicephalus sanguineus* ticks have high preferences for sheep and goats, while Amblyomma species are large, three-host parasites confined to the tropics and subtropics [10, 13]. Many reports over recent years have documented tick-borne pathogens in Egypt [14] that might co-exist within the same tick [1]. Our understanding of these agents’ interactions within the tick and vertebrate host remains poorly defined and represents a current knowledge gap [15].

Babesiosis and theileriosis are the most frequently tested TBPDs affecting ruminant animals, leading to major financial losses. Treatment involves specific drugs such as atovaquone-azithromycin or imidocarb, depending on the species and host [16]. Babesia spp. affecting livestock (e.g., *B. bovis*, *B. bigemina*, *B. ovis*) are not considered zoonotic. Only certain species, such as *B. microti*, *B. divergens*, and *B. duncani*, are known to cause human babesiosis. They are small, pear-shaped, round, or oval parasites that result in hemolytic anemia, potentially leading to organ failure and death in immunocompromised hosts. Babesia infection can be subclinical, cause self-limited febrile illness, resemble malaria, or rapidly progress to death, depending on species and host factors [17]. Previous studies in Egypt frequently detected *B. bovis* and *B. bigemina*, showing similar prevalence, and a few datasets also detected other species (e.g., *B. ovis* and *B. occultans*) [18].

Theileria is a hemoprotozoan parasite responsible for tropical theileriosis in the bovine population, which causes substantial economic losses to the livestock sector. Tropical theileriosis is a cancer-like disease that affects ruminants and is characterized by enlarged lymph nodes, fever, swollen eyelids, profuse lachrymation, anemia, jaundice, and occasionally mortality [19]. The genetic diversity of the protozoan aids in the parasite’s ability to evade the host’s immune response and ensures its long-term survival in host animals [20]. Of these, *T. annulata* and *T. parva* were more predominant subspecies than others in Egypt [21]. Although the populations of water buffaloes and cows in Egypt are nearly the same, buffaloes have received little attention concerning TBPs [22].

Diagnostic strategies for babesiosis and theileriosis include the microscopic examination of characteristic intraerythrocytic organisms on Giemsa-stained thin smears. Microscopic analysis was unable to distinguish between closely similar organisms or reliably identify Babesia and Theileria infections in carrier animals, especially when parasitemia was extremely low [23]. Therefore, molecular tools continue to be essential for identification of novel pathogens and differentiation [24]. The molecular characterization studies primarily involve selective targets of *Babesia 18 S rRNA* and *T. annulata tams1* genes that are highly specific for Babesia species and *T. annulata*, respectively, with no cross-reactivity with other spp [25]. Phylogenetic and sequence analysis showed that *T. annulata* 18 S rRNA isolates shared homology and phylogeny with other isolates from Asia and Europe [25].

New Valley is the largest governorate in Egypt, comprising roughly half of Egypt’s area, in southwest Egypt, at 24°32′44″N 27°10′24″E (Wikipedia, the free encyclopedia). It has a hot, dry climate; especially, the high summer temperatures create a suitable environment for various tick species [22]. High tick activity can occur throughout the year except in cold months [1]. Tick control is an important strategy for combating tick-borne parasites (TBPs). The continuous application and prolonged incorrect use of acaricides on farms resulted in acaricide-resistant tick populations and created a potential future problem for controlling TBPs [26]. This study aims to determine the prevalence and genotypic diversity of Babesia spp. and *T. annulata* in cattle, sheep, and goats in the New Valley Governorate, Egypt, and to identify associated tick vectors through microscopy, PCR, and phylogenetic analysis.

## Materials and methods

### Study area

The New Valley Governorate in Egypt’s Western Desert is the largest area, covering 440,098 km², representing approximately 43.6% of the total area of Egypt. It is a depression between the Nile, northern Sudan, and southeastern Libya, and its coordinates are between 24° 32′ 44° N and 27° 10′ 24° E. The three main oasis coordinates of the New Valley Governorate are Kharga, 25° 26’ 10.79” N, 30° 33’ 17.99” E; Dakhla, 25° 30’ 59.99” N, 29° 09’ 60.00” E; and Farafra, 27°03′30″N, 27°58′12″E, besides others, are in depressions that fall below the average surface of the desert. There was a severe drought and groundwater depletion because of low rainfall. Livestock farmers and veterinarians are concerned about tick-borne diseases, which cause significant suffering in the region. Veterinary authorities are working to eliminate these diseases through various methods, including pesticides and traditional ways.

### Study design

We conducted the present study from September 2023 to July 2024. It began with a spot survey to identify ticks and tick-borne parasites. We administered a questionnaire to gather information about all animals, newly introduced animals, and their origins on the farm, and also to inquire about regular antiprotozoal medication use. Bedouins and herders in the study area prefer to keep one selected male from each animal species in a herd for insemination. Therefore, most samples were taken from females. Their ages ranged from months to 10 years, with the majority of goats and sheep being between two and four years old and cattle between five and 10 years old. Therefore, these factors were neglected in the comparison. Microscopical examination and DNA amplification of PCR-based assays targeting genes specific to examined parasites identified samples. Then, we sequenced and performed phylogenetic analyses on some of the PCR-strong positivity-generated fragments to identify the dominant parasite subspecies strains, and we recorded them in the GenBank database.

### Examined animals

We randomly selected 321 live animals (89 cattle, 55 goats, and 177 sheep) of all sexes (276 females and 45 males) and aged between 9 months and 10 years between September 2023 and July 2024. Animals belong to 27 mixed flocks; each flock contains 15 to 250 animals. The sample size of each examined species (cattle, goats, and sheep) was determined by practical constraints from private farms and animal traders but was not less than 5% of the total animals. All animals were healthy except 74, which showed symptoms such as abortion, diarrhea, fever, enlargement of lymph nodes, deficiency of vitamins and mineral salts, nasal discharge, and a few had corneal opacity. The Egyptian Authority Program mandated regular vaccination of the animals. The basal diets consisted of a mixture of wheat straw and concentrates offered twice a day, in the morning and evening, with free access to water.

### Tick collection and morphological identification

We collected 269 adult ticks from 203 infested animals out of 321 studied animals in the study area as previously described [27]. We preserved the ticks in 70% alcohol and transported them to the laboratory for morphological identification. Then, we mounted the tick specimens on glass slides, counted them, and identified them morphologically using a stereomicroscope, following the taxonomic key by [28] and the recent valid names of the genus and species. The key identification features of the ticks are color, size, shape of mouthparts, scutum, anal groove, festoon, punctuation, and legs.

### Sample collection and parasites

Each animal had five milliliters of blood drawn from its jugular vein using sterile, clean Vacutainer tubes that contained ethylene diamine tetra-acetic acid (EDTA). We divided it into two parts: (1) for microscopic analysis and (2) preserved in the freezer at −20 °C until needed for DNA extraction for PCR amplifications. From every sample, two thin blood films were left to air dry, fixed for five minutes with absolute methyl alcohol, and then stained for thirty minutes with 15% Giemsa stain. They were identified using the characteristics outlined by [29] after being inspected under a light microscope using an oil immersion lens (x1000).

### Molecular detection and genotyping of tick-borne *Babesia* and *Theileria*

#### DNA extraction and primer selection

Genomic DNAs were extracted from 321 blood samples using the DNeasy Blood and Tissue Kit (Qiagen, Hilden, Germany) according to the manufacturer’s instructions. Briefly, 200 µL of the sample or blood was treated for 10 min at 56 °C with 10 µL of proteinase K and 200 µL of lysis buffer. Following incubation, the lysate was mixed with 200 µL of 100% ethanol, in accordance with the manufacturer’s instruction. A total of 100 µL of elution buffer was used to elute the nucleic acids after the washing step. Spectrophotometer estimated recovery and purity of each DNA sample (NanoDrop^®^ ND-1000, PeqLab, Erlangen, Germany) and then stored at −20 °C for use.

Primer sequences 5’−3’ were F, GTAACCTTTAAAAACGT, and R, GTTACGAACATGGGTTT, sensitive and specific for the *T. annulata tams1 gene* that amplifies 721 bp [30], and F, GTCTTGTAATTGGAATGATGGTGAC, and R, ATGCCCCCAACCGTTCCTATTA, sensitive and specific for the *Babesia 18 S rRNA gene* that amplifies 340 bp [31]. They were supplied by Metabion (Planegg, Germany).

#### PCR amplification

PCR-based assays were subjected to detect blood parasites using those species-specific primers and conducted in a total volume of 25 µL. Each reaction contained 12.5 µL of commercial Mastermix (Takara, Tokyo, Japan), 20 pmol of each primer, ~ 25 ng of genomic DNA, and sterile water in an automatic DNA thermocycler (Bio-Rad T100, Hercules, USA). For each PCR run, a negative control (water) without template DNA and positive controls, including DNA from known reference clinical strains, was donated from the Institute of Animal Health (Dokki, Giza, Egypt). The PCR process involved one cycle of primary denaturation at 94 °C for 5 min., followed by 35 cycles of second denaturation at 94 °C for 30 s. Different annealing temperatures were 55 °C for 40 s for *T. annulata tams1* and 56 °C for 40 s for *Babesia 18 S rRNA* genes. The polymerization step was one cycle at 72 °C for 10 min. Twenty µl of each PCR product sample, negative control, and positive control were loaded onto the gel and electrophoresed using a voltage gradient of 5 V/cm on a 1.5% agarose gel (Applichem GmbH, Darmstadt, Germany) in 1× TBE buffer at room temperature. The fragment size was determined using the GeneRuler 100 bp Ladder (Fermentas, Darmstadt, Germany). A gel documentation system (Alpha Innotech, San Leandro, CA, USA) was used to display the gel, and computer software was used to evaluate the data. The specific bands are indicative of present parasites.

### Sequencing and phylogenetic analyses

Primers, nonspecific bands, and other contaminants were eliminated prior to DNA sequencing.

To guarantee sufficient quantities and purity of the PCR products, as well as to produce high yields, the PCR product was quantified using Nanodrop (UV-Vis spectrophotometer Q5000, Waltham, MA, USA) [32]. For gene sequencing, we selected 11 highest positivity products based on gel band intensity, six for *Babesia* sp. and five for *T. annulata*, with amplicon sizes of 340 and 721 bp, respectively, and purified them directly using a QI-quick PCR product extraction kit (Qiagen Inc., Valencia, CA). They sequenced in both directions on an Applied Biosystems 3130 automated DNA sequencer (ABI, 3130, USA), and a ready-to-use BigDye Terminator V3.1 cycle sequencing kit (Perkin Elmer/Applied Biosystems, Foster City, CA) (Cat. No. 4336817) was used for sequencing. The sequencing chromatograms were not manually curated to correct ambiguous bases. Single-nucleotide polymorphisms (SNPs) categorized disparities between the reference sequences found in GenBank and the PCR results of the genes under investigation. The phylogenetic trees were created by the Neighbor-joining model with bootstrap support (1000 replications), and similarities between isolates were determined using the Maximum Likelihood test in Molecular Evolutionary Genetics Analysis Version 6.0 (MEGA6) software [33].

### Statistical analysis

Using SPSS V20.0, we analyzed the survey results and categorized samples by infested animals, tick samples, seasons, and parasites. The chi-square test was applied to compare the differences in results. All statistics were considered significant at *P* ≤ 0.05. The nucleotide sequences were aligned with existing sequences of corresponding parasites in the GenBank databases using BLAST programs [34] and databases of the NCBI (National Center for Biotechnology Information, Bethesda, MD, USA) (www.blast.ncbi.nlm.nih.gov/Blast.cgi*).* It was done using the CLUSTALW multiple sequence alignment programs, version 1.83 of the MegAlign module of Lasergene DNAStar software Pairwise.

## Results

### Tick infestation and seasonal dynamics

Of the 321 investigated animals, 203 (63.2%) were infested with one or two tick species, and 269 ticks were collected. The survey results (Table 1) indicate substantial differences (*P* < 0.0001) in the presence of ticks on animals across the year. During the rainy season (December and January), the number of collected ticks was 41/269 (15.2%) but reached 228/269 (84.8%) during the dry season (March-October). *Rhipicephalus annulatus*, the cattle tick accounting for 182/269 (67.7%), had higher prevalence on cattle, followed by *Hyalomma excavatum* 48/269 (17.8%). *Rhipicephalus sanguineus* preferred sheep and goats and was prevalent in 39/269 (14.5%). *H. dromedarii* has not been detected on the examined animals.

**Table 1:** Frequency and distribution of tick-infested animals, according to tick species and season of collection

	Examined animals	Infested animals	Ticks species			Total ticks	Dry Seasons	Rainy seasons
			*R*. annulatus	H. excavatum	*R*. sanguineus			
Cattle	89	71	97	10	8	115	96	19
Goats	55	47	64	22	17	103	89	14
Sheep	177	85	21	16	14	51	43	8
Total	321	203(63.2%)	182(67.7%)*	48(17.8%)	39(14.5%)	269	228(84.8%)**	41(15.2%)

Seasonal significant differences were highly significant** between numbers of ticks in dry and rainy seasons (*P*<0.001), while tick species differences were significant* (*P*<0.05) between different tick species

### Microscopical and molecular detection of pathogens

**Fig. 1:**
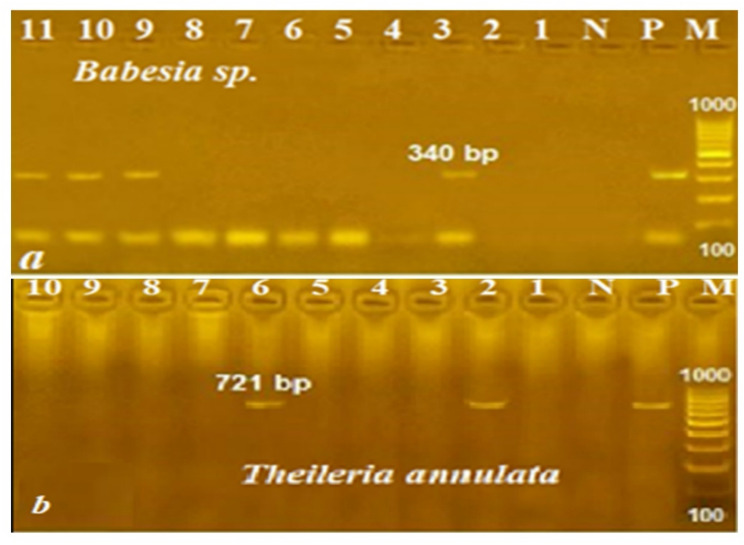
PCR amplification products obtained from genomic DNA of *Babesia* (**a**) and *Theileria annulata* (**b**) of ruminants using primers specific for *Babesia 18 S rRNA* (340 bp) and *Theileria annulata tams1* gene (721 bp). Lanes M: molecular weight standards (100 bp); Lanes N and P: negative and positive controls

PCR was more sensitive and specific than microscopy when parasitemia was extremely low in chronic infections. Results shown in Table 2 and demonstrated in Fig. 1 revealed that out of 321 examined animals, 71 (22.1%) harbored *Babesia*, whereas 53 (16.5%) harbored *T. annulata* by microscopic examination. Cattle were the most susceptible to infection with *Babesia* (28.1%), and goats were the most susceptible to *T. annulata* (25.5%), while sheep had the lowest rates of infections (22.03%, 15.3%) for the two parasites. PCRs increased the percentage of infections to 32.7% for babesiosis and 22.1% for theileriosis using cPCR-based assays. It confirmed the greater susceptibility of cattle to babesiosis (35.9%) and goats to theileriosis (29.1%) than sheep, which had the least to examine for parasites (30.5%). Both methods detected mixed infections in the same animals, but PCR detected some mixed infections with higher microscopy in other animals and could distinguish between trophozoites in mixed infections since they looked so similar. Mixed infections were present in 25 (7.79%) and 43 (13.4%) animals by microscopical examination and PCR assays, respectively.

**Table 2:** Microscopical and molecular survey of Babesia species and *Theileria annulata* in the studied ruminants

Host	Microscopical examination							Molecular survey						
	No.	Babesia spp.		Theileria annulata		Mixed infections		No.	Babesia spp.		Theileria annulata		Mixed infections	
		+Ve	%	+Ve	%	+Ve	%		+Ve	%	+Ve	%	+Ve	%
Cattle	89	25	28.1	12	13.5	6	6.74	89	32	35.9	21	23.6	10	11.24
Goats	55	7	12.7	14	25.5	7	12.7	55	19	34.5	16	29.1	11	20.0
Sheep	177	39	22.03	27	15.3	12	6.78	177	54	30.5	34	19.2	22	12.43
Total	321	71	22.12	53	16.5	25	7.79	321	105	32.7	71	22.1	43	13.4%

*No.* number of examined animals, +Ve = infected

### Genotyping and phylogenetic analysis

**Fig. 2:**
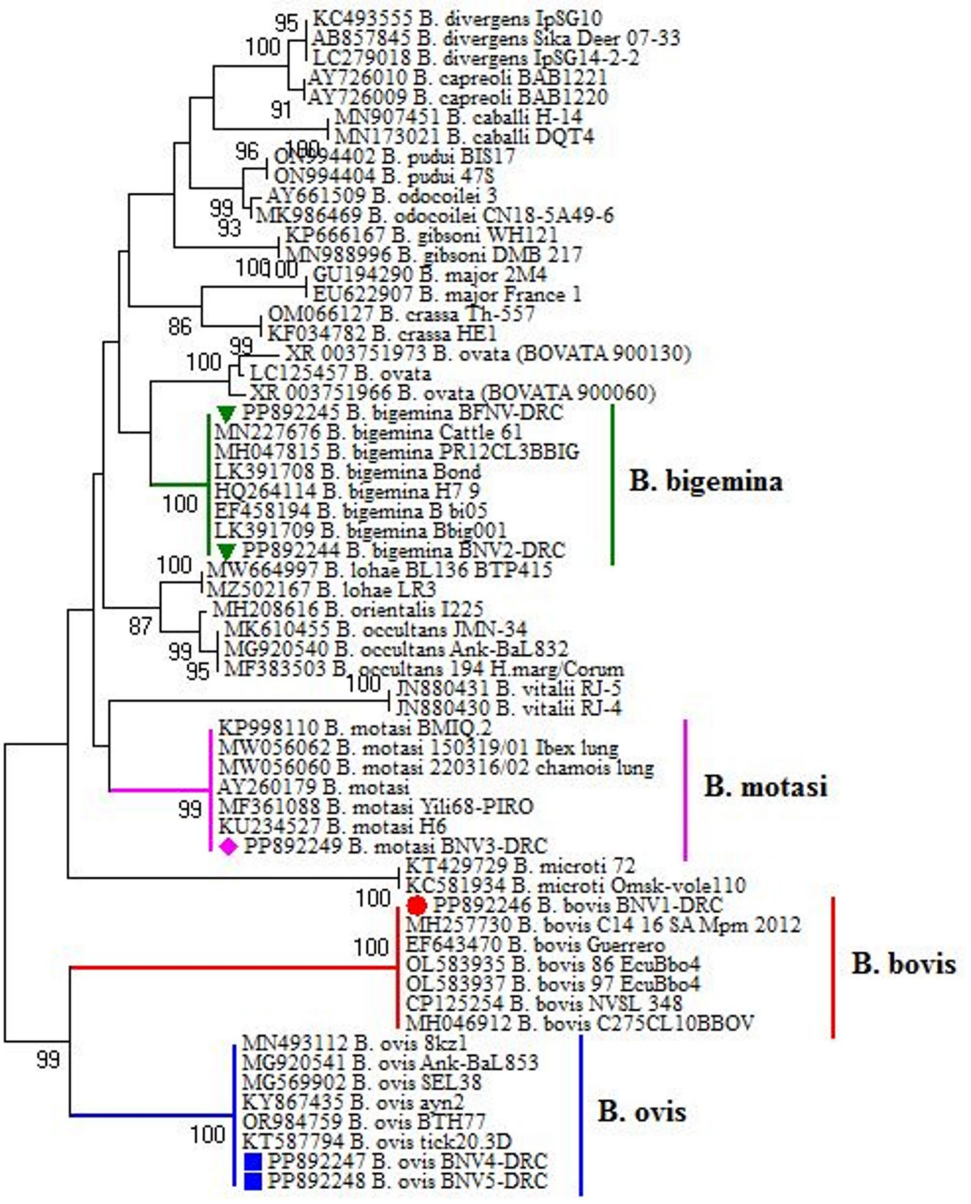
Phylogenetic tree of *B. bigemina*, *B*. *motasi*, *B. bovis*, and *B. ovis* genotypes inferred from the partial sequences of the *18 S rRNA* gene for submitted and referenced isolates of Babesia species. Our accession numbers are colored, and the tree was computed by Neighbor-joining (MEGA 6.0 software)

**Fig. 3:**
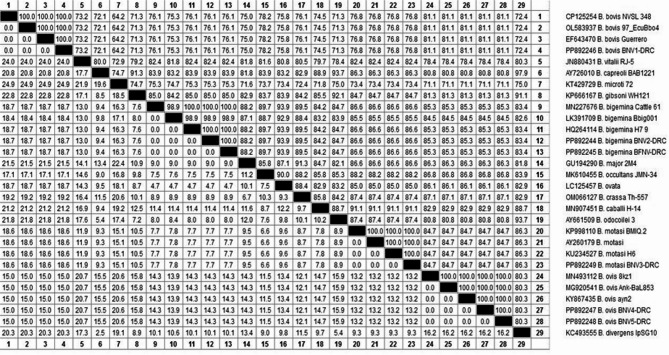
The percent of identity and genetic distances for our submitted* Babesia *speciesbased on the the *Babesia 18S rRNA gene* that amplifies 340 bp. *Our accession numbers are followed by DRC (Desert Research Center)*

In the current study, four subspecies of *Babesia* were identified from six gene sequences, categorized into four classes: *B. bigemina* in cattle (*n* = 2), *B*. *motasi* in sheep (*n* = 1), *B. bovis* in cattle (*n* = 1), and *B. ovis* in goats (*n* = 2), as shown in Table 3 and in the phylogenetic tree in Fig. 2. They are introduced in the GenBank database under accession numbers PP892244 and PP892245 (as *B. bigemina*), PP892249 (as *B. motasi*), PP892246 (as *B. bovis*), and PP892247 and PP892248 (as *B. ovis*), distributed in two clusters. Their identities to GenBank references range from 83.4% to 100% (for *B. bigemina*), 71.3% to 100% (for *B. bovis*), 84.7% to 100% (for *B. motasi*), and 80.3% to 100% (for *B. ovis*), and divergence from 7.6 to 18.7 (for *B. bigemina*), 0.0 to 24 (for *B. bovis*), 0.0 to 13.2 (for *B. motasi*), and 0.0 to 13.2 (for *B. ovis*) as illustrated in Fig. 3.

**Table 3:** The GenBank database of the present isolates and the most closely identities of preserved isolates

	Acc No.	Country	Isolation source/host	Reference
*B. bigemina*	PP892245	Egypt	Cattle	The present study
	PP892244	Egypt	Cattle	The present study
	MN227676	Egypt	Cattle	[35]
	MH047815	USA	Bovine	[36]
	HQ264114	USA	White-tailed deer	[37]
	EF458194	Australia	*Bos taurus*	[38]
*B. motasi*	PP892249	Egypt	Sheep	The present study
	KP998110	Iraq	Sheep	[39]
	MW056062	Italy	*Rupicapra rupicapra*	[40]
	MW056060	Italy	*Rupicapra rupicapra*	[40]
	AY260179	Germany	Sheep	[41]
	MF361088	China	*Haemaphysalis punctata*	[42]
	KU234527	United Kingdom	*Haemaphysalis punctata*	[43]
*B. bovis*	PP892246	Egypt	Cattle	The present study
	MH257730	South Africa	Cattle	[44]
	EF643470	Mexico	Not identified	[45]
	OL583935	Ecuador	Not identified	[46]
	OL583937	Ecuador	Not identified	[46]
*B. ovis*	PP892247	Egypt	Goat	The present study
	PP892248	Egypt	Goat	The present study
	MN493112	Turkey	Not identified	[47]
	MG920541	Turkey	*Rhipicephalus bursa*	[48]
	MG569902	Turkey	Equine	[49]
	KY867435	Turkey	*Rhipicephalus sanguineus*	[50]
	OR984759	Japan	Not identified	[32]
	KT587794	Palestine	Ticks	[51]

**Fig. 4:**
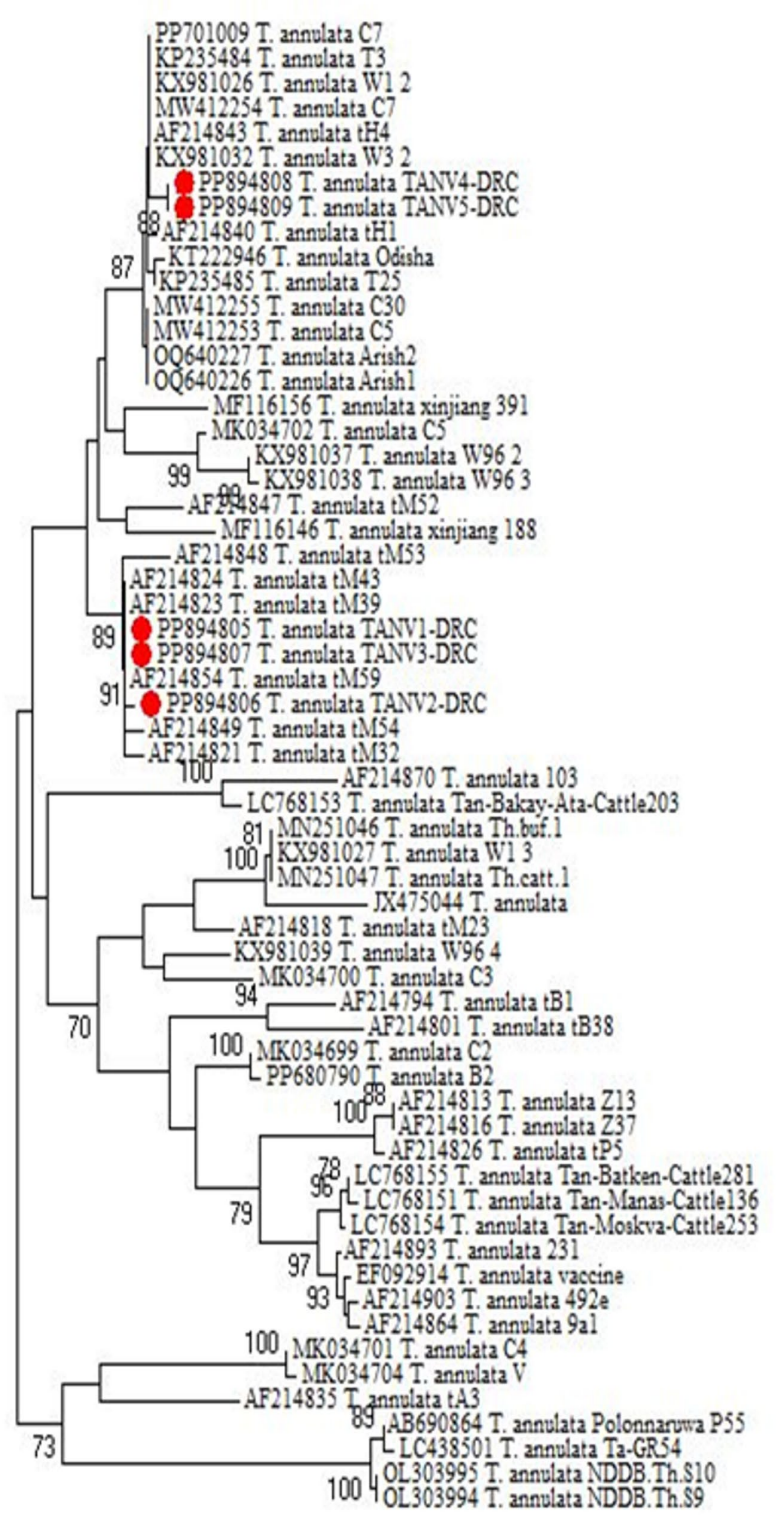
Phylogenetic tree of* Theileria annulata *genotypes inferred from the partial sequences of the *Theileria annulata* *tams1* gene for submitted and referenced isolates. Our accession numbers are in red, and the tree was computed by Neighbor-joining (MEGA 6.0 software).

**Fig. 5:**
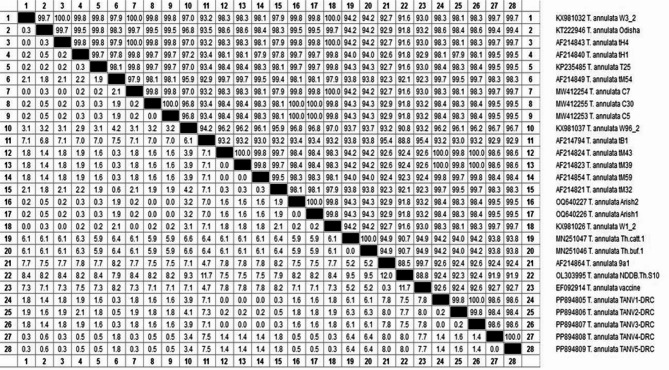
The percent of identity and genetic distances for our submitted *Theileria annulata* based on the *Theileria annulata tams1* gene that amplifies 721 bp. *Our accession numbers are followed by DRC (Desert Research Center)*

The current study also revealed that the five submitted isolates of *T. annulata* were distributed into two clades within a cluster (Fig. 4). It showed no significant genotypic differences between them. They were recorded in the GenBank database under accession numbers PP894805 and PP894806 from cattle, PP894807 from sheep, and PP894808 and PP894809 from goats (Table 4). Sequence analysis revealed close relationships with corresponding GenBank sequences from cattle, sheep, and goats isolated from India, the United Kingdom, Pakistan, Mauritania, and Egypt. The percent identities range from 98.4 to 100%, and the strains had a divergence of 0.0 to 1.6 between each other and 0.0 to 8.0 from others in GenBank, as shown in Fig. 5.

**Table 4:** The most closely related isolates of present *Theileria annulata* to that preserved in the GenBank database

	Accession No.	Country	Isolation source/host	Reference
*Theileria annulata*	PP894808	Egypt	Goats	The present study
	PP894809	Egypt	Goats	The present study
	PP701009	India	Not identified	[52]
	KX981026	United Kingdom	Not identified	[53]
	KX981032	United Kingdom	Not identified	[53]
	KP235484	India	*Hyalomma marginatum*	[54]
	MW412254	Pakistan	Cattle	[55]
	OQ640226	Egypt	*Camelus-dromedarius*	[56]
	OQ640227	Egypt	*Camelus-dromedarius*	[56]
	PP894805	Egypt	Cattle	The present study
	PP894806	Egypt	Cattle	The present study
	PP894807	Egypt	Sheep	The present study
	AF214843	Mauritania	Calf	[57]
	AF214824	Mauritania	Calf	[57]
	AF214823	Mauritania	Calf	[57]
	AF214854	Mauritania	Calf	[57]

## Discussion

Hard ticks are blood-feeding ectoparasites that infest humans and animals, inducing severe infectious diseases. The present study should update the epidemiological data concerning the prevalence of tick-borne protozoan parasites in New Valley Governorate on the borders of Libya and Sudan, in the southwest of Egypt. The importation of animals infected with TBDs from endemic countries, like Sudan and Ethiopia, are quarantined in Egypt before being transported to slaughterhouses or animal markets in populated areas, where the risk of transmission of ticks and TBDs is much higher. Ticks, such as *R. annulatus* and *H. excavatum*, are reported as prevalent in some Egyptian localities due to favorable conditions and animal importation. These ticks significantly impact the cattle, buffalo, sheep, goat, camel, and horse industries, causing severe anemia, weight loss, and skin damage [2]. The present study found that *R. annulatus* was the dominant tick on cattle, while *H. excavatum* was dominant on cattle in New Valley by the integrated analyses of [58]. It also revealed the absence of *H. dromedarii* because of the lesser extent of rearing local or imported camels, the main host for ticks.

*Babesia* and *Theileria*, two related genera of hemoprotozoan parasites, are worldwide and infect free-living animals. Their prevalence is second to trypanosomes, and the geographical distribution of Babesia and Theileria infections is highly variable and depends on the tick vector’s distribution [24, 59, 60]. The current study provides additional information on Babesia infections that will help in developing strategies for controlling the disease. Based on the observed animal health conditions, most animals appeared to be healthy, while the others showed clinical signs including persistent weakness, lethargy, depression, fever, inappetence, jaundice, anemia, and weight loss. Controlling babesiosis is necessary because of its potential negative impact on milk and meat production in this area. The primary control strategies for this tick-borne disease are mostly based on active disease surveillance through periodic screening programs, treatment of afflicted animals, and prevention of disease dissemination by eradicating vector ticks. Nomads in the study area used repellents like permethrin and DEET, besides injectable ivermectin, that are effective against ticks but require frequent reapplication.

Active infection cases are diagnosed using classic Giemsa-stained blood smears that rely on identifying the characteristic intraerythrocytic organisms present, although more sophisticated diagnostic techniques, including molecular and serological approaches, are available. In the current study, the initial microscopical diagnoses ended at the genus level, exposing two parasite genera, including *Babesia* and *Theileria*, with comparable results. The exposure of animals to ticks carrying multiple hemoparasite species in various settings and the tick species may explain mixed infection rates found on the animals, as described by [60]. There was a correlation between the two results of the two tests, but PCR was superior to microscopy in the lab. In addition, PCR differentiated between trophozoites in mixed infections and was more sensitive and specific than microscopy, especially in low parasitemia in chronic infections. Despite that, the microscopic diagnosis was partially reliable and suitable for the field. The level of infection varies from one parasite to another; however, the studied animals had virtually the same rates but lower rates of babesiosis, with no significant differences (*P* ≥ 0.05).

The prevalence observed in the present study (22.1% microscopy; 32.7% PCR for Babesia) falls within the range reported in other Egyptian governorates but is higher than rates noted in southern regions and lower than those in the Delta [58–66]. Comparable studies detected *B. bigemina* in 25 (92.6%) calves and 21 (84%) cows in Brazil [67], 11.11% in Tunisia [68], 18.8% in Pakistan [69], 78.5% in Portugal [70], and 26.7% in the Philippines [71]. Although the examined ruminants grazed together, sheep had the lowest infection rate of the others. It may be attributed to the length and span of their hair, which could limit tick bites, making it more difficult for them to have a blood meal and, therefore, transfer those parasites and this is consistent with what we saw during our field visits.

Concerning theileriosis, the infection rates with *T. annulata* in the three investigated hosts are consistent with some of those reported in Egypt, but they were lower than previously published for other governorates of the Delta, Upper Egypt, and South Sinai in Egypt [72–79]. Ecological factors specific to each region, the distribution of ticks, hygiene management, or geographical distribution may explain the differences between our findings and those of prior studies. The infection rate in our study is relatively similar to that obtained in domestic animals in other countries. However, the PCR assay identified *T. annulata* at rates of 22.1% in the present study compared to 23.4% in Tanzania [80], 37.8% in eastern Turkey [81, 82], and 32.6% in Tunisia [83]. Goats were more likely to be infected by *T. annulata* because frequent contact with vegetation and vector habitats in oasis environments may facilitate tick exposure, explaining the higher infection rates observed. To prevent economic loss for farmers have been included, we informed the farmers and veterinarians in the study area about the laboratory results of blood parasites to treat infected animals.

The population structures of many protozoan parasites are predominantly clonal [84]. Surveys of some vector-borne parasite species, such as Babesia sp., have shown high levels of genetic diversity [85]. There is little information regarding the diversity of Babesia species detected in ruminants in the study region, which was only centered on the El-Kharga Oasis and slaughtered animals. Despite the low prevalence of infection per species, certain areas of Dakhla Oasis are still active and have the potential to transmit different blood parasites through transportation. Babesia was identified in all districts during the current examination, and the molecular and phylogenetic research revealed that several domestic animals (goats, sheep, and cattle) are infected with different Babesia species. They had previously been reported in domestic animals in various districts across Egypt [24, 86]. These similar results could be explained by the fact that these parasites’ vectors are widely distributed throughout the Egyptian deserts, as well as the movement of animals from one governorate to another, which is facilitated by breeding, commerce, and agricultural operations, which explain the variety of parasites present in domestic animals.

In the present study, the two identified *B. bigemina* were introduced in the GenBank database under accession nos. PP892244 and PP892245, revealing high conservation of isolates identical with four identified strains: MN227676 from Egypt [35], HQ264114 and MH047815 strains from the USA [36, 37], and EF458194 from Australia [38]. The two identified sequences, *B. ovis*, grouped with four identical strains from cattle and one from South Africa under accession number MH257730 [44], as well as three isolates from South America: EF643470 from Mexico [45] and OL583935 and OL583937 from Ecuador [46]. The third sequence, designated as *B. motasi* with GenBank accession number PP892249, isolated from sheep, clustered with six identical strains, two from Asia and four from Europe. It was associated with one isolate of *Haemaphysalis punctata* from China (MF361088; [42]) and one isolate from sheep in Iraq (KP998110; [39]). It was identical and grouped with two strains of *B. motasi* under accession nos. MW056062 and MW056060 that were isolated from *Rupicapra rupicapra* in Italy [40], one isolated from sheep in Germany (AY260179) [41], and the other isolated from Haemaphysalis punctata in the United Kingdom (KU234527) [43]. The fourth sequence, identified as *B. ovis* with GenBank accession numbers PP892247 and PP892248 generated for goats in the current investigation. They were more similar to the strains isolated from Turkey and recorded in GenBank under accession numbers MG569902 from equine [49] and ticks MN493112 [47], MG920541 [48], KY867435 [50], KT587794 from Palestine [51], and OR984759 from Japan [32].

Regarding Theileria, the *Tams-1 gene* was utilized to verify and identify the genetic diversity of potential novel genotypes of *T. annulata* by running BLAST and phylogenetic analyses. The two analyses revealed that the newly isolated strains from the study area clustered together in two clades within a single cluster, closely related to other strains in the GenBank database. Also, there are no significant differences in the genotypes of *T. annulata* isolates from three different continents based on *T. annulata* ITS sequences; thus, it does not exhibit geographic specificity when comparing those isolates with those sourced from other countries. Sequence analyses also revealed that the present *T. annulata* genotypes (accession numbers PP894805 and PP894806 from cattle, PP894807 from sheep, and PP894808 and PP894809 from goats) are identical and closely related to others preserved in the GenBank sequences. They reveal percent identity ranges from 98.4 to 100% within each other and from 91.6 to 100% with the most related isolates with accession numbers of KP235484 and PP701009 from India [52, 87]; XY981026 from the United Kingdom [53]; MW412254 from Pakistan [55]; AF214821, AF214824, AF214843, and AF214854 from Mauritania [57]; and OQ640227 and OQ640226 from Egypt [56]. The divergence between the present strains ranged from 0.0 to 1.6 and from 0.0 to 8.0, with others in the GenBank database suggesting low diversity between local strains and others.

This study is limited by its regional scope (New Valley Governorate) and the inability to assess clinical status, parasite load, or acaricide resistance. Future studies should integrate seasonal sampling, vector monitoring, and longitudinal surveillance.

## Supplementary Information

Supplementary Material 1.

## Data Availability

Data availability the datasets supporting the conclusions of this article are included within the article (and its additional files).
