# Supplementary material for: Detection and genotypes of piroplasms affecting ruminants in the New Valley Governorate, Egypt
Source: BMC Vet Res. 2025 Nov 15;21:669. doi: 10.1186/s12917-025-05101-3 (PMC12619443; doi:10.1186/s12917-025-05101-3)
Supplement: Supplementary file 1 — Supplementary Material 1. [file 12917_2025_5101_MOESM1_ESM.docx]

Response : This is the original figures for Theileria annulata and Babesia (I only substituted the number of samples during the laboratory work to new numbers from 1 to 10 (for the journal)

**
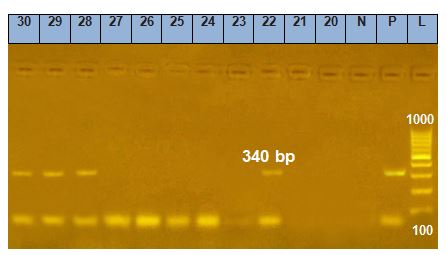
**

**
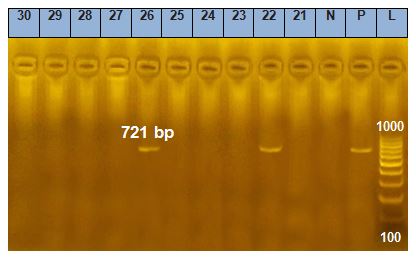
**

Fig.1 PCR amplification products obtained from genomic DNA of *Babesia* and *Theileria annulata* of ruminants using primers specific for *Babesia 18S rRNA* (amplify 340 bp) and *Theileria annulata* *tams1* gene (amplify 721 bp). Lanes M: molecular weight standards (100 bp); Lanes N and P: negative and positive controls


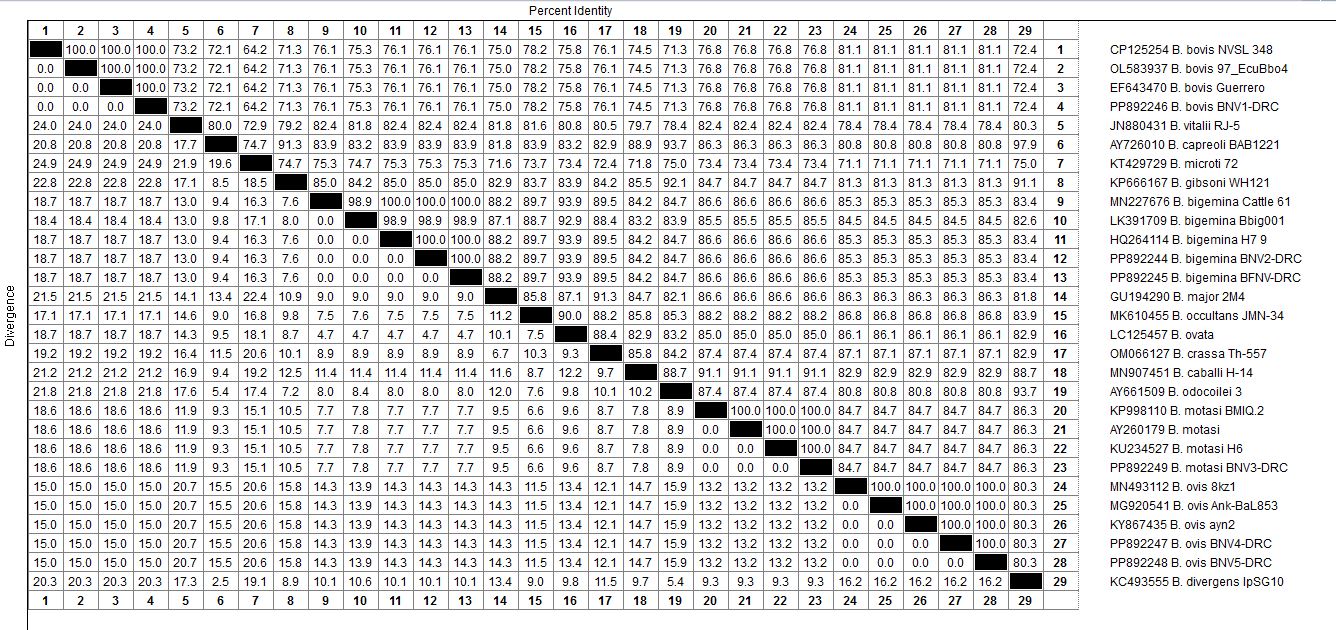


Fig. 4 The percent of identity and genetic distances for our submitted *Babesia* species based on the the *Babesia 18S rRNA gene* that amplifies 340 bp. *The accession numbers of our submitted isolates are followed by DRC.*


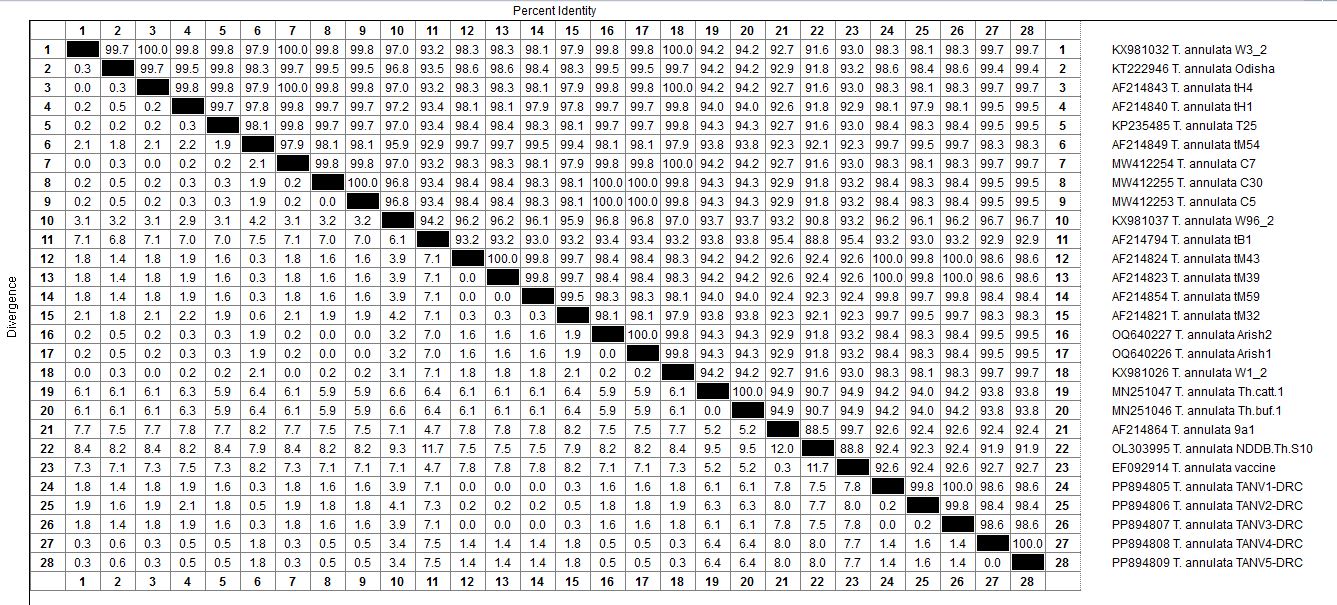


Fig.5 The percent of identity and genetic distances for our submitted *Theileria annulata* based on the *Theileria* *annulata* *tams1* gene that amplifies 721 bp. *The accession numbers of our submitted isolates are followed by DRC.*
